# Supplementary material for: Geochemical constraints on bacteriophage infectivity in terrestrial environments
Source: ISME Commun. 2023 Aug 18;3:78. doi: 10.1038/s43705-023-00297-7 (PMC10439110; doi:10.1038/s43705-023-00297-7)
Supplement: Supplementary file 2 — Supplementary Figures [file 43705_2023_297_MOESM2_ESM.pdf]

Supplementary Figures:

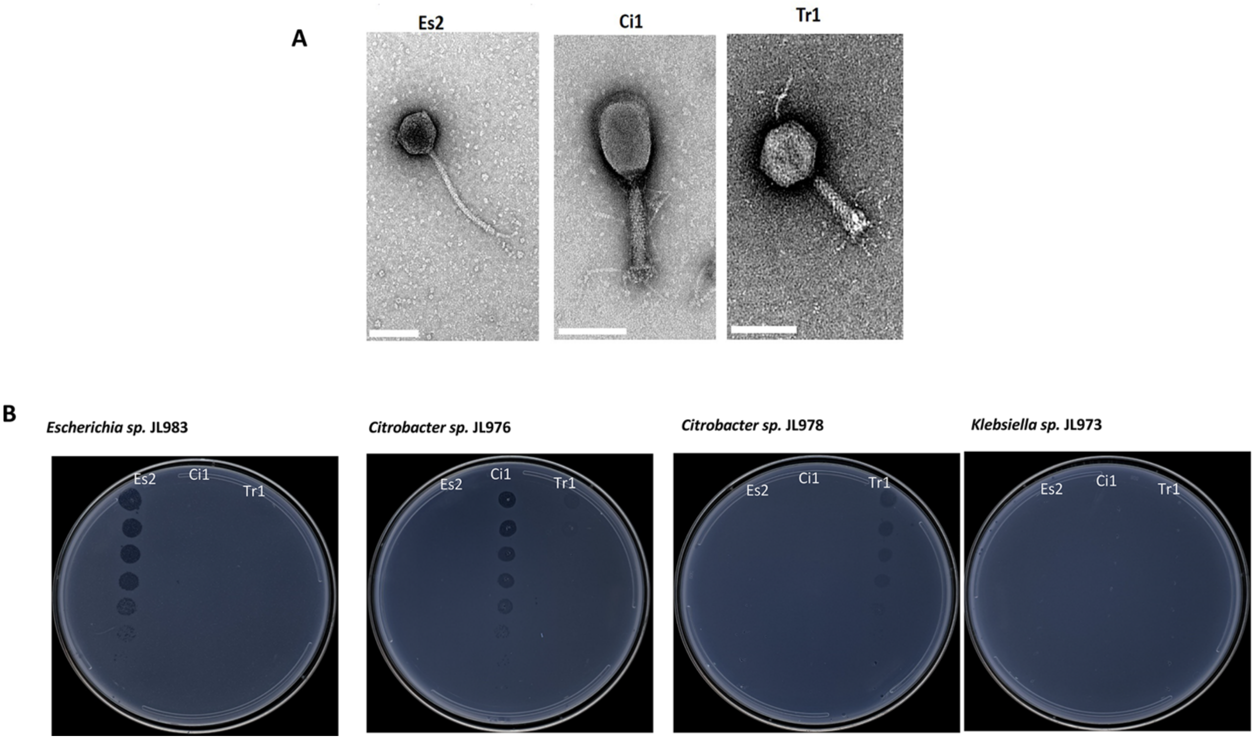

Figure S1. Phages in cocktail formulated to inhibit DNRA in freshwater nitrate reducing enrichment (FN) cultures. **A.** Transmission electron micrographs of ES2, Ci1 and Tr1 phages. The scale bar denotes 100 nm **B.** Plaque assays demonstrating specificity of phages for dominant *Enterobacteria* in the FN culture. ES2 is specific for *Escherichia* JL 983, Ci1 is specific for *Citrobacter sp.* JL 976 and Tr1 is specific for *Citrobacter sp.* JL 978.

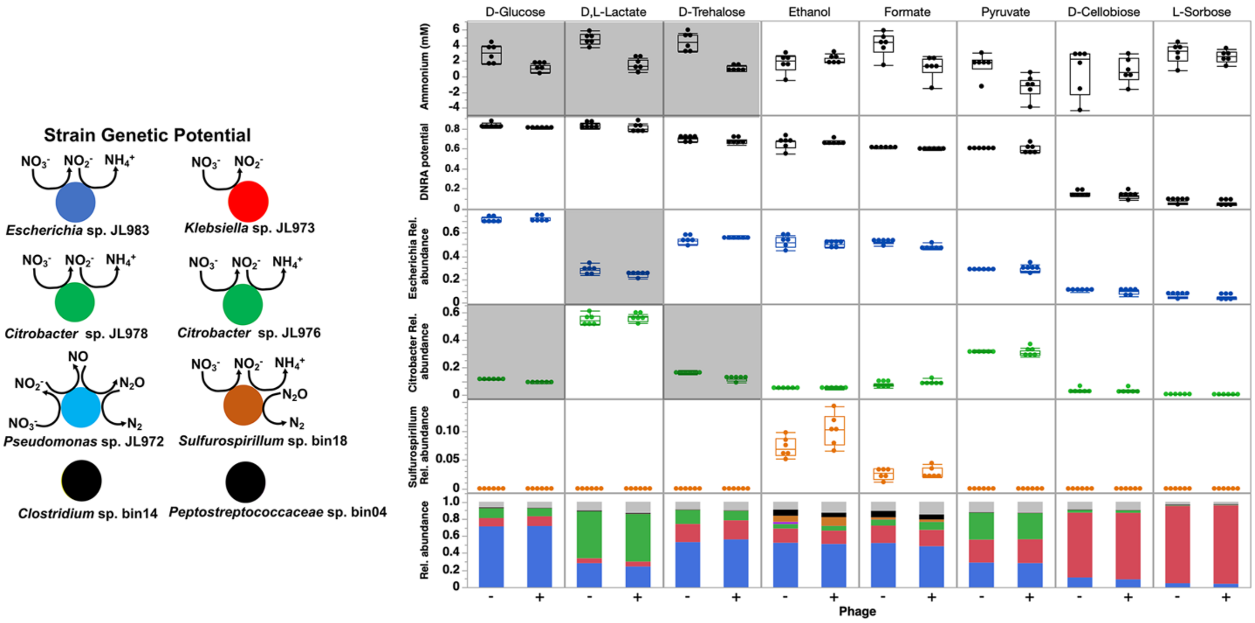

Figure S2. Ammonium production, DNRA genetic potential and relative abundances of dominant strains in the FN microbiome on selected carbon sources. In box plots: Box and whiskers represent interquartile range. Shaded panels indicate significant differences (ANOVA, $p<0.05$) between minus (-) and plus (+) phage conditions. In stacked bar plot: Dark Blue = *Escherichia*, Green = *Citrobacter*, Red = *Klebsiella*, Orange = *Sulfurospirillum*, light blue = *Pseudomonas,* Black = Gram-positive fermenters, Gray = other strains.

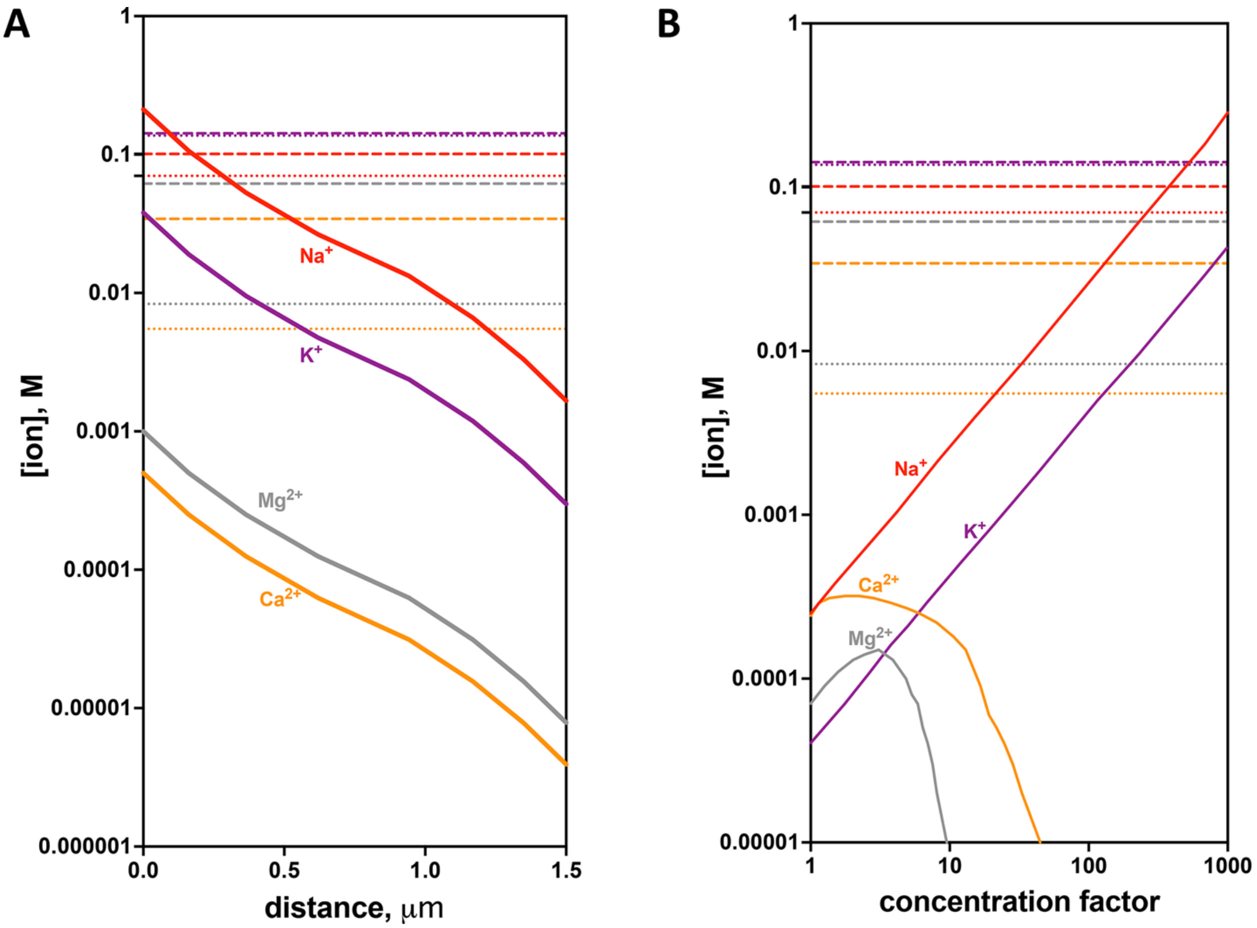

Figure S3. Temporal and spatial control of phage infectivity by ion thresholds in terrestrial ecosystems. **A.** Major cytoplasmic ion concentrations as a function of diffusion distance from lysed *E. coli* cells compared with EC_{50} concentrations required for T4 (dash), or MS2 (dotted) phage infection. **B.** Major ion concentrations in a typical Sierra Nevada spring water during evaporation as a function of concentration factor compared with phage EC_{50} . Colors are consistent between EC_{50} and ion concentration lines. (colors and symbols are the same as panel A).
